# A rare location of thyroglossal duct cyst in a newborn

**DOI:** 10.11604/pamj.2018.31.104.14777

**Published:** 2018-10-10

**Authors:** Amel El Korbi, Rachida Bouatay, Jihène Houas, Karim Ben Ameur, Khaled Harrathi, Jamel Koubaa

**Affiliations:** 1 ENT Department, Fattouma Bourguiba Hospital of Monastir, University of Monastir, Tunisia; 2 Healthcare Research Unit UR12SP41, Tunisia; 3 Neonatal Department, Maternity and Neonatal University Center, Unversity of Monastir, Tunisia

**Keywords:** Newborn, thyroglossal cyst, tongue, surgery

## Abstract

Thyroglossal duct cyst of the tongue is a rare entity. Occurrence in the anterior part of the tongue is exceptional. We report in this paper a rare case of thyroglossal cyst of the anterior part of the tongue, discovered in a five-days-old newborn at delivery. Images have shown a cystic mass with homogenous liquid content. A transoral complete resection of the lesion was performed, with no postoperative complication. The histological analysis confirm the diagnosis of lingual thyroglossal duct cyst. There were no recurrence with a follow-up of eight months.

## Introduction

Thyroglossal duct cyst (TGDC) represents the most common congenital midline mass. It results from remnant epithelial rests of the thyroid gland during it migration throughout the development from the tongue base [1]. Its location in the tongue is a rare entity, observed in 0.6% to 3% of cases at the base of the tongue [2]. Occurrence in the anterior part of the tongue is exceptional [3]. Symptoms are made of dysphagia, obstruction of upper airways and even death have been reported [4, 5]. The thyroglossal duct cyst of the tongue can be confused with others cystic masses ie: ranula, dermoid cyst, monocystic lymphangioma. The radiological investigation (ultrasonography, tomography and magnetic resonance imaging) are very helpful for the diagnosis. However, in some cases we could not be sure of the diagnosis only on the histological analysis of the resected specimen. We report in this paper a case of a neonatal thyroglossal duct cyst of the anterior part of the tongue managed with a transoral resection without complication. The length of disease-free follow-up was of 8 months.

## Patient and observation

**Figure 1:**
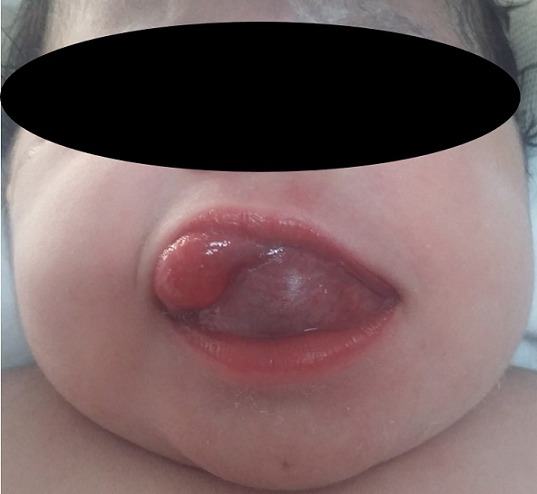
Lingual mass at clinical examination

**Figure 2:**
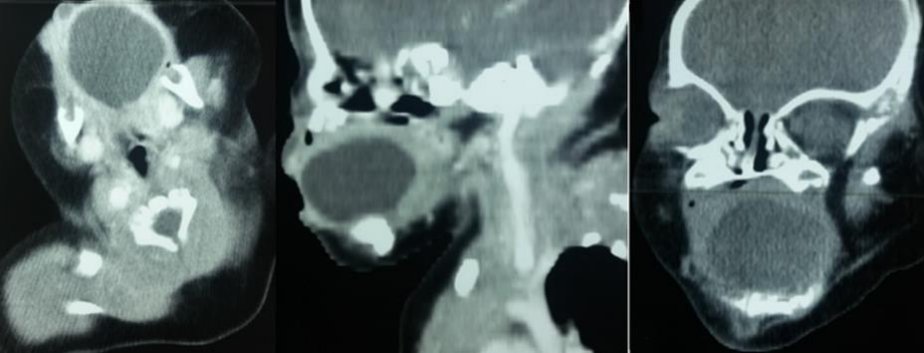
Axial, sagittal and coronal enhanced CT scan showed a large thin-walled cystic mass, which located in the anterior part of the tongue with a homogenous liquid-density content

**Figure 3:**
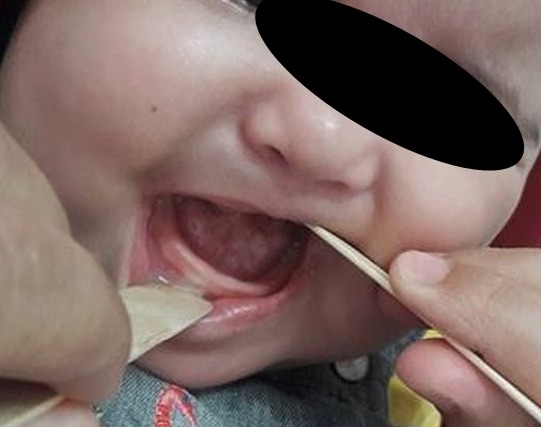
Clinical aspect eight months after transoral resection

A five-days-old newborn was referred to our department for an enormous lingual mass discovered at delivery. It was a normal 3,900 grams female, born by caesarean section performed because of failure to progress in labor. The Apgar score was 9/10 at 1 and 5 minutes of life. The ENT examination found a renitent mass of the tongue which measure 4 centimetres of large with a difficulty of suction but no respiratory signs (Figure 1). The ultrasonography has shown a cystic thin-walled lesion of 37 x 30 mm, with liquid-liquid level. The thyroid gland was normally located. A computed tomography had been performed to better characterize the lesion. It has shown a 36 mm large thin-walled cystic mass, which located in the anterior part of the tongue with a homogenous liquid-density content (Figure 2). A puncture with aspiration of the cyst content in object to reduce its size was done then a transoral complete resection of the lesion were performed with no post-operative complication and the infant was discharged two days later. The histological analysis of the specimen has confirmed the diagnosis of lingual thyroglossal duct cyst. There were no recurrence with a follow-up of eight months (Figure 3).

## Discussion

Lingual thyroglossal duct cyst (LTGDC) is a rare location, which occur in 0.3-3% of cases, and it is often interest the posterior third of the tongue [2]. The literature reports few cases of occurrence in the anterior part of the tongue [3]. The TGDC can clinically appear at any age and at any site of the pathway migration of the thyroid from caecum foramen to pyramidal lobe [6]. Untreated, the TGDC can cause an upper airways obstruction that can occasionally leads to fatal asphyxia [4, 5]. Effectively, the prenatal diagnosis is possible and the neonatal care and management should be prepared to avoid an eventually a neonatal asphyxia [2]. Images (ultrasonography, CT scan) affirm the cystic nature of the lesion but it can be confused, especially in newborns, with a ranula, bronchogenic cyst, dermoide cyst, lymphangioma, so that MRI should be performed when it is possible [2]. In our case, we have decided not to do an MRI because of additional anaesthesia. Sistrunk procedure is the gold standard of a TGDC resection with the lowest recurrence level [2]. Meanwhile, some authors have adopted more conservative techniques in management of TGDC of the tongue which leads to less complication [2]. Marsupialization is one of techniques adopted with a high rate of recurrence [2, 6]. Bai *et al.*had treated six of nine patients with LTGDC with a needle puncture. They observed recurrence in two cases (3). The transoral complete resection without recurrence was reported [2, 4, 5]. Actually, the transoral robotic system resection of LTGDC seems to be a rather efficient method in order to reduce the rate of complications and hospital stay [1]. In our case, because the lingual cyst was anteriorly located, we have decided to perform a transoral approach after a needle aspiration. We suggest that the resection of the hyoid bone shall be discussed when the lingual thyroglossal cyst is anteriorly located, especially since some authors have published cases of thyroglossal cyst managed without Sistrunk procedure with no cases of recurrence. When the location is the base of the tongue, we think that a cervical approach is justified.

## Conclusion

Diagnosis of LTGDC in newborn is not usually evident. Image is essential in order to locate exactly the lesion, MRI when it is possible, is preferred because its sensitivity in soft tissues lesions characterization. Surgical approach depends on the location in the tongue and proximity to the hyoid bone.

## Competing interests

The authors declare no competing interests.
